# Community-scale big data reveals disparate impacts of the Texas winter storm of 2021 and its managed power outage

**DOI:** 10.1057/s41599-022-01353-8

**Published:** 2022-09-24

**Authors:** Cheng-Chun Lee, Mikel Maron, Ali Mostafavi

**Affiliations:** 1 Texas A&M University, College Station, TX USA; 2 Mapbox, San Francisco, CA USA

**Keywords:** Science, technology and society, Complex networks

## Abstract

Aggregated community-scale data could be harnessed to provide insights into the disparate impacts of managed power outages, burst pipes, and food inaccessibility during extreme weather events. During the winter storm that brought historically low temperatures, snow, and ice to the entire state of Texas in February 2021, Texas power-generating plant operators resorted to rolling blackouts to prevent collapse of the power grid when power demand overwhelmed supply. To reveal the disparate impact of managed power outages on vulnerable subpopulations in Harris County, Texas, which encompasses the city of Houston, we collected and analyzed community-scale big data using statistical and trend classification analyses. The results highlight the spatial and temporal patterns of impacts on vulnerable subpopulations in Harris County. The findings show a significant disparity in the extent and duration of power outages experienced by low-income and minority groups, suggesting the existence of inequality in the management and implementation of the power outage. Also, the extent of burst pipes and disrupted food access, as a proxy for storm impact, were more severe for low-income and minority groups. Insights provided by the results could form a basis from which infrastructure operators might enhance social equality during managed service disruptions in such events. The results and findings demonstrate the value of community-scale big data sources for rapid impact assessment in the aftermath of extreme weather events.

## Introduction

As extreme weather events, such as floods, hurricanes, earthquakes, and winter storms occur more frequently and with higher intensity due to climate change, life-threatening circumstances and damage of critical infrastructure (e.g., power, water services, transportation, and communications systems) will impact human systems and the environment (Fan and Mostafavi, 2019; Kim and Hastak, 2017; Lu et al., 2018; Panteli and Mancarella, 2015; Coronese et al., 2019; Stocker, 2014; Mostafavi, 2018). Such disaster events have a disproportionate impact on vulnerable subpopulations (i.e., those of lower socioeconomic status, minorities, and the elderly) (Fothergill et al., 1999; Donner and Rodríguez, 2008; Hong et al., 2021). Thus, understanding the disparate effect of the past events on vulnerable subpopulations is critical for development of equitable mitigation plans and to prepare response strategies to adapt to other disasters (Kryvasheyeu et al., 2016; Morss et al., 2011).

Communities are not homogenous entities; in the case of a disaster, residents’ awareness, needs, responses, and tolerance vary as functions of inequalities of race, ethnicity, income, gender, and age during and in the aftermath of disasters (Bolin and Kurtz, 2018; Elliott and Pais, 2006; Reid, 2013; Peacock et al., 2014). Elliott and Pais (Elliott and Pais, 2006) analyzed survey data collected from more than 1,200 survivors of Hurricane Katrina and found strong differences in the hurricane’s impact as a function of race and class. Using tax appraisal data, (Peacock et al., 2014) assessment of long-term trends in housing recovery for Hurricanes Andrew and Ike showed that low-income areas tend to suffer more damage and recover more slowly. Results of an investigation of empirical data regarding service disruptions and impacts on critical infrastructure by Coleman et al. (Coleman et al., 2020) after Hurricane Harvey revealed that less advantaged socioeconomic households, racial minorities, and households with younger residents reported decreased ability to withstand disruptions. In sum, the literature revealed existing inequalities in the context of disaster management. Studies of extreme cold weather situations, however, are scarce. The US National Oceanic and Atmospheric Administration defines a winter storm as a life-threatening combination of freezing precipitation and dangerous wind chill (“National Oceanic and Atmospheric Administration”, 2022). According to the declared disasters database of US Federal Emergency Management Agency, only two severe winter storms were federally declared emergencies after 2010 (“Federal Emergency Management Agency”, 2022). One was the winter storm in Georgia and South Carolina in 2014, which resulted in at least 25 deaths and economic losses of more than $750 million USD (Podlaha & Bowen, 2014). The other was Winter Storm Uri in 2021 in the southern United States. Texas, one of the most heavily impacted states, suffered at least 146 deaths due to hypothermia and an estimated cost of $195 billion USD attributed to blackouts (Hellerstedt, 2021; Ferman, 2021). Thus, Winter Storm Uri affords a unique opportunity to examine to extent to which the impacts of power outages, burst pipes, and food inaccessibility were disproportional across subpopulations of different races, ethnicities, and incomes.

Extreme weather events may necessitate that power infrastructure owners and operators implement managed outages to cope with the surge in demand in the cases of winter storms, extreme heat, or to prevent wildfires. Despite the growing practice of managed power outages during extreme weather events, little evidence exists regarding the extent to which these managed outages and power restoration are implemented in an equitable manner (Mitsova et al., 2018). In addition, only limited studies explored the population reaction to power outages (Rubin and Rogers, 2019). This limitation is partly due to the inability of researchers to access fine-resolution data related to the extent and duration of outages for subpopulations. By using power outage data during Hurricane Irma, (Mitsova et al., 2018) found that strong hurricane winds are only one of many factors affecting the duration of power outages. The results show that customers served by different utility cooperatives, as well as those with social vulnerabilities, experienced longer power outages during Hurricane Irma. Yet, despite efforts by the authors to obtain fine-grained and high-resolution power outage data, power outage data for the Texas winter storm of 2021 is unavailable; therefore, we address this limitation by harnessing digital trace data to obtain proxy measures. In addition to the use of digital trace data for examining the extent of power outages, we also used other community-scale data sources for rapid impact assessments related to burst pipes and disrupted access to food to reveal how residents react to a power outage.

Social media platforms have made available large sets of de-identified data upon which researchers have relied to evaluate disaster impact in recent years. For example, social media data have been employed to sense the impact of community disruption (Zhang et al., 2020), assess disaster footprints and damage (Resch et al., 2018), categorize disasters for response (Ragini et al., 2018), and map disaster locations (Fan et al., 2020a). Social media data, however, can be affected by factors such as income, population size, population composition, and minority population ratio (Xiao et al., 2015). The results from evaluation of social media may be biased due to unbalanced user populations among socioeconomic groups and affected areas. In addition, not every piece of social media information is geo-coded; for example, only 1% to 2% of tweets have geospatial information; (Fan et al., 2020b; Morstatter et al., 2013) this underrepresentation may give biased results and which could stymie the tracking of population activity and behavior. On the other hand, several methods have been proposed to evaluate the community impact of crisis events using new technology that provides large-scale digital trace data (Hong et al., 2021; Podesta et al., 2021). Detailed spatiotemporal data reveal unique insights into the interdependence of disaster problems, human activity, and urban mobility. The new insights can help us assess the impact of disaster more accurately (Zhang et al., 2020), respond to disaster promptly (Li et al., 2021a), and enhance preparedness for future events (Fan et al., 2021).

In this study, we used digital trace and crowdsourced datasets to evaluate the impact of the 2021 winter storm in Texas. Population activity can be derived from cell phone signal densities. Higher cell phone signal densities may indicate more population activities in a location; thus, cell phone signal densities can be aggregated into an activity index as a proxy for population activity. A few studies have incorporated activity index into disaster-related research; for example, (Yuan et al., 2022) identified human activity features for flood impacts, and (Gao et al., 2021) discovered early indicators for COVID-19. Similarly, digital trace data can provide a reliable proxy for the extent and duration of power outages. The level of cell phone signal activity would fluctuate during a power outage due to, for example, evacuation of a place of residence in the event of power loss or lack of power to charge cell phones. In addition to digital trace data from cell phone activities, the time series of population visits to points of interest (POIs) is a commonly used indicator of population activities. Analysis of visits to POIs, such as restaurants, gas stations, and grocery stores, reveals insights into patterns of population movement and destinations during a disaster event. Several studies have assessed the impact or determined the signal of disasters, such as the COVID-19 pandemic (Li et al., 2021a, 2021b), Hurricane Harvey (Podesta et al., 2021), and Hurricane Irma (Juhasz and Hochmair, 2020), by implementing POI visit data. The fluctuations in POI visits could provide reliable insights regarding disrupted access to critical facilities, such as grocery stores and restaurants.

The objectives of the paper are to (1) categorize the responses to infrastructure failure (e.g., managed power outages) and to assess the impacts (e.g., burst pipes and food inaccessibility) of an unusual extreme weather event, and (2) reveal potential disparate impacts associated with a population’s income, race, and ethnicity to an unusual event by employing community-scale big data from various sources. The study used Texas Winter Storm Uri, in February 2021, as a case study to fulfill the objectives of the paper.

## Materials and methods

**Fig. 1:**
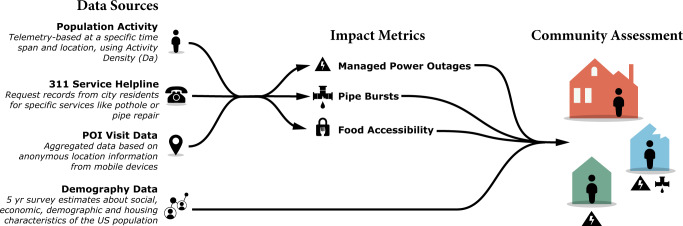
Schematic of the study to assess the impact of Winter Storm Uri. We gathered and aggregated data of population activity, point-of-interest (POI) visits, 311 service helpline calls, and demographic information from several data sources to assess community impacts related to the winter storm-induced power outages, burst pipes, and food inaccessibility.

This study gathered and aggregated data related to population activity, point-of-interest (POI) visits, 311 service helpline calls, and demographic information from several data sources. We collected and integrated data to assess community impacts related to the winter storm-induced power outages, burst pipes, and food inaccessibility (Fig. 1). All data were aggregated to the census-tract level to be comparable. The study area, period, and details of each data source are discussed in the following sections.

### Study area and period

The study collected and analyzed data from Harris County, Texas, which encompasses the Houston metropolitan area. From February 13 through 17, 2021, a severe and icy winter storm, Winter Storm Uri, affected most of North America and the entire state of Texas. Harris County was one of the most adversely affected areas in the state. The winter storm reached Harris County in the late evening of February 14, 2021, bringing snow and sub-freezing temperatures. Residents of Harris County were warned of the impending snow and low temperatures starting February 10, 2021. On the afternoon of February 12, 2021, disaster declarations were in effect in all 254 Texas counties. (The National Weather Service issued winter weather alerts to more than 154 million people in the United States on February 15, 2021 (Almasy et al., 2021).) The impact of the storm was especially significant on Texas residents, who are not experienced with cold weather, snow, or icy conditions. Historic records, both temperature and duration, were broken, and at least 146 people passed away in Texas due to hypothermia (Hellerstedt, 2021; Freedman et al., 2021; Mulcahy, 2021). Traffic was affected due to icy and slick road conditions. A 133-car pileup on Interstate Highway 35W in Fort Worth caused six fatalities (Cappucci, 2021). The state’s electricity infrastructure was overwhelmed due to heating demand, necessitating rolling blackouts to avoid collapse of the entire electrical grid. Nearly 4.5 million Texas homes and businesses lost power during the peak of this crisis (Mulcahy, 2021). The estimated cost of the blackouts was at least $195 billion USD (Ferman, 2021). In the study area of Harris County, more than 90% of residents lost power, and about 65% of the residents experienced water outages at some point during the winter storm (Watson et al., 2021). For this study, we obtained data from January and February 2021 to conduct the analyses. The January 2021 data, averaged by grouping data from the same day of the week in successive weeks, served as the baseline for the assessments. The study then compared data from February 2021 with the baseline data to understand the impact of disaster preparedness, response, and recovery. Data from different sources were integrated and aggregated at the census-tract level in Harris County, which has 786 census tracts.

### Data sources

#### Population activity

Attempts by the authors to obtain fine-resolution power outage data from publicly accessible power outage records and the electricity owner and operator company were unsuccessful. Therefore, this study assessed the impact of power outages during the winter storm by analyzing telemetry-based population activity provided by Mapbox. Mapbox collects users’ cell phone location from applications that use the Mapbox Software Development Kit (SDK) and aggregates, normalizes, and anonymizes the geography information to estimate population activity. Population activity data from Mapbox has been used in several studies to shed light on population responses during disaster events (Yuan et al., 2022; Gao et al., 2021; Farahmand et al., 2022). Since telemetry-based population activity would decrease during blackout periods, the data could provide a reliable proxy for the extent and duration of power outages during the winter storm. For example, telecommunication base stations could not transmit signals, cable modems and home Wi-Fi routers could be nonfunctional, and cell phones batteries could be without a means of recharging. Mapbox aggregated raw cell phone information by time span and geographic unit and normalized this data to a baseline period, January 11 to 17, 2021, for this study. After Mapbox aggregated and normalized the raw data, activity index (*A*), a scaling factor was calculated by dividing the cell phone device counts of geographic units over the 99.9th percentile across all geographic units in the baseline period. A geographic unit is a point in a spatial-resolution grid representing certain areas in the study areas. The 99.9th percentile of cell phone device counts across all geographic units represents the highest population activity of the study areas excluding some potential outliers. In other words, if a geographic unit received 0.5 at a specific time span, that designation indicates that the cell phone device counts of the geographic unit are 50% of the highest cell phone device counts of all geographic units at its baseline period. The more users located in a geographic unit at a time span, the higher the population activity. Mapbox applied two anonymization processes to protect users’ privacy. First, in geographic areas with only a few counts below minimum requirements, the counts would be dropped. Secondly, slight random noise was applied to the existing counts. While the data was derived from cell phone information, data may not exist in every geographic unit at all times. For example, a museum with tourist visits during the day may close at night, resulting in reduced population activity and thus has insufficient nighttime data to be aggregated. Some places may lack data if the visit counts are too few at a particular time. The geographic unit of the activity index provided by Mapbox is in a 100-m by 100-m spatial-resolution grid; the temporal resolution is 4 h. The number of geographic units within a census tract depends on the land area and the population activity level of the census tract. In this study, the median number of geographic units having activity index within a census tract is about 30 every day. To make the data comparable, the activity index was aggregated and used to calculate the activity density (Da) of each census tract (ct) at each time period (*t*). The calculation is:

$${\mathrm{Da}}\left( {{\mathrm{ct}},\,t} \right) = \sqrt {\frac{1}{N}\mathop {\sum }\limits_{u = 1}^N A_{u,t}^2}$$

where, Da(ct*, t*) is the activity density at time *t* in census tract ct, *A*_*u,t*_ is the activity index at time *t* of geographic unit *u*, and *N* is the number of geographic units within census tract ct. By calculating activity density for every census tract in Harris County and every time period between February 1 through February 28, 2021, the spatiotemporal pattern of population activity can be identified.

#### Point-of-interest data

SafeGraph, which partners with several mobile applications to obtain anonymous location data from mobile devices, provided point-of-interest data used in this study. The aggregated POI data includes basic information about places, such as location name, latitude, and longitude, address, and business category denoted by standard North American Industry Classification System (NAICS) code. The POI data provides the visit counts to a POI every day over the analysis period.

To evaluate the impact of the winter storm, this study identifies two types of POIs for analyses: grocery stores (NAICS code: 445110) and restaurants (NAICS code: 72251). The data for grocery stores can reflect changes in terms of daily needs and food inaccessibility. The fluctuation of restaurant visits can be extended to identify the food inaccessibility of communities. We applied visit data at the census-tract level to isolate visit changes during the winter storm; however, some residents living close to the boundary of census tracts may frequent restaurants or grocery stores in neighboring census tracts. To account for this consideration, we applied a one-mile buffer to all census tracts when aggregating visit data to the census-tract level. In other words, the aggregated visit data of a census tract would include the POIs within a one-mile distance from the boundary of the census tract.

#### 311 Service Helpline

The 311 Houston Service Helpline is a consolidated call center where city residents can report non-emergency concerns, such as traffic signal malfunction, water leaks, sewer concerns, potholes, and garbage container problems. Residents can also request information on city services. The details of each 311 record include start and finish time, latitude and longitude, and the type of service request. In this study, the 311 data was used to assess the impact of burst pipes. Water-related service requests were filtered to collect 311 records related to burst pipes. The data were further aggregated to the census-tract level based on the latitude and longitude of each request. In this manner, the count of cases of burst pipes in each census tract on each day in February 2021 can be calculated and used for analysis.

#### Demography

**Fig. 2:**
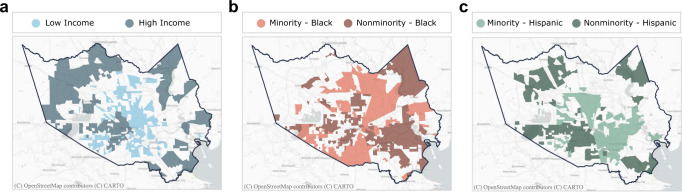
Locations of the high- and low-income groups and racial/ethnic minority and nonminority groups. High/low income (**a**) and minority/nonminority groups for Black (**b**), and Hispanic (**c**) populations were identified in terms of the top 25% and bottom 25% levels, respectively, in Harris County.

All demographic and household socioeconomic data used in this study were retrieved from the 2019 American Community Survey 5-year estimates data (2015–2019) administrated by US Census Bureau and aggregated by census-tract level. The census tracts within the top or bottom 25% of data were classified as the high- and low-income and the racial/ethnic minority and nonminority groups (Fig. 2). The low-income group represents the census tracts with relatively low median household income. The racial minority group represents the census tracts with a relatively large percentage of Black residents. The ethnic minority group represents the census tracts having a relatively high ratio of persons of Hispanic origin. The demographic data thus obtained when compared with the assessment of the impact of the winter storm can be applied to understand the disparity in experience between various income, racial, and ethnic groups during the disaster.

### Statistical analysis

#### Kruskal–Wallis test

To examine the difference in impacts on the minority and nonminority populations, this study used the Kruskal–Wallis test, or one-way ANOVA on ranks, due to the non-normality of the residuals on a study population defined by income, race, and ethnicity. The Kruskal–Wallis test is a nonparametric method without an assumption of normal distributions of the residuals. Instead, the shapes of all groups need to be identical. Accordingly, the null hypothesis of the Kruskal–Wallis test is that the medians of the two groups are equal. The alternative hypothesis is that the median of one population group is different from the median of the other group. In other words, a significant test result indicates that the population median values of the minority and nonminority populations, in terms of such as income and Hispanic population, are different.

#### Spatial autocorrelation

Spatial autocorrelation was conducted by using Global Moran’s *I* to understand whether spatial dependency exists in the results. Moran’s *I* ranges between –1 and 1; a positive Moran’s *I* indicates attributes of objects are similar to other nearby objects. In contrast, a negative Moran’s *I* suggests objects are dispersed in a spatial area. To determine neighbors of an area, this study used queen contiguity weights as the spatial weights to represent spatial relationships among study areas. The queen contiguity weights determine neighbors by identifying a polygon sharing an edge or a vertex with another polygon. To understand the significance of the Moran’s *I* values, this study used the test approach of Monte Carlo simulation, which samples a reference distribution of Moran’s *I* values by randomly assigning attribute values to polygons several times and comparing the observed Moran’s *I* value with the reference distribution. This study generated the reference distribution of Moran’s *I* from 999 permutations and computed a pseudo *p*-value to determine the significance of the Moran’s *I* value. A significant test result suggests that attributes are not randomly distributed across the study area.

### Trend classification

In addition to the statistical analysis and comparisons of spatial patterns, an agglomerative hierarchical clustering algorithm (Hastie et al., 2008; Day and Edelsbrunner, 1984) was implemented to classify neighborhood visit trends and also to understand the pattern response to the impact of the winter storm by residents of a census tract. The classification of POI visitation trends enables evaluation of the variation of impacts across subpopulations. Agglomerative clustering is a commonly used hierarchical clustering method in machine learning and data mining to group data points into clusters based on their similarity or distance. The algorithm determines clusters in a greedy manner and starts by treating each data point as a single cluster. At each iteration, the distances between every pair of clusters in the dataset are calculated. Also, similar clusters are merged with neighboring clusters based on proximity until all clusters are combined into one cumulative cluster containing all data points. The study uses Euclidean distance to determine similarity of different clusters and uses Ward’s linkage criterion, which minimizes variance within clusters, to determine the clusters to be merged at each iteration. Unlike clustering algorithms such as k-means clustering, which have randomness in the initial steps, the agglomerative hierarchical clustering algorithm considers every data point at every iteration. This algorithm has been used in disciplines such as physiology (Ray et al., 2020; Steiger et al., 2019), transportation (Pasupathi et al., 2021), and disaster resilience (Hong et al., 2021) to provide critical insights by grouping big data.

## Results

After data processing and aggregation of all data at the census-tract level, we assessed community impacts due to the Texas winter storm in three aspects: power outages, burst pipes, and food inaccessibility. In the absence of granular power outage data, the impact of power outages caused by the winter storm was inferred based on population activity data. During the extreme cold weather, electricity demand increased while the supply decreased significantly due to vulnerable power-generating facilities in Texas not being winterized nor designed to operate in unseasonable cold (McCullough et al., 2021). The Electric Reliability Council of Texas (ERCOT) had no choice but to shut down the partial power grid to avoid complete failure. It is important to understand, however, whether these outages were experienced in an equitable manner by different subpopulations in terms of race, ethnicity, and income. In addition, this study assessed the impact of burst pipes related to freezing gleaned from 311 service helpline calls in Harris County. Owing to the cold weather and limited preparedness beforehand, reports of frozen and burst water pipes were widespread in Harris County. Food accessibility was also critically limited during the winter storm due to dangerous road conditions. This study relied on data documenting visits to restaurants and grocery stores to assess the impact of the winter storm on food inaccessibility. Through this analysis, results based on community-scale big data from various resources were used to address two research objectives: (1) to quantify and to evaluate the spatial patterns of the impacts of and response to the winter storm on the community; (2) to evaluate the extent of disparities in impacts experienced by low-income and racial/ethnic minority subpopulations. The following sections present and discuss the results in more detail.

### Impact of power outages

**Fig. 3:**
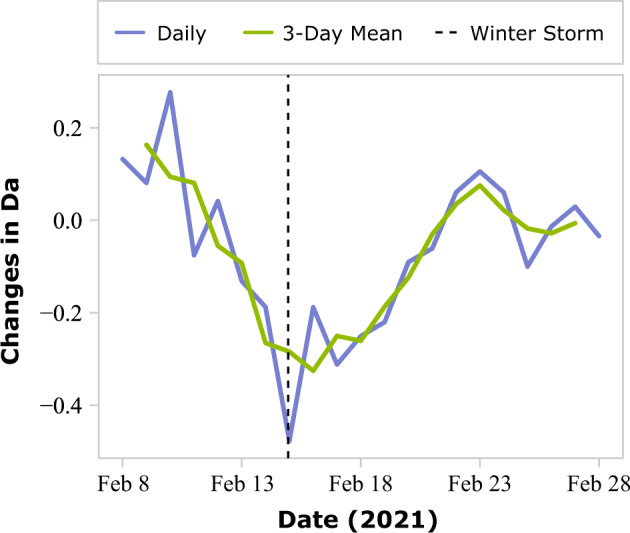
Activity density (Da) changes of a census tract in Harris County compared to its baseline period during the winter storm. The census tract experienced an almost 50% decrease in population activity on February 15, 2021, when the winter storm reached, and recovered to the normal states with a few fluctuations about February 22, 2021.

Without electricity, the functioning of most appliances, as well as communications, water, and transportation may be disrupted. Thus, assessing the impact of power outages and understanding which populations and areas are affected is critical for orderly disaster recovery. Despite efforts by the authors to gather fine-grained and high-resolution power outage data to enable impact and disparity assessment, publicly accessible power outage records are not granular enough to be used for this purpose. Thus, telemetry-based population activity data served as a reliable proxy for examining the extent of power outages. A consequence of a power outage may be loss of internet connectivity or persons traveling from their houses to find a power source in an unaffected area. Activity index, a telemetry-based index provided by Mapbox, was aggregated and used to calculate activity density (Da) at the census-tract level. More than half of the census tracts in Harris County did not register enough activity to calculate activity density during the night; therefore, only data documenting activity between 8:00 a.m. and 8:00 p.m. were included for analysis. Figure 3 demonstrates the activity density changes of a census tract in Harris County compared to its baseline period. The census tracts experienced an almost 50% decrease in population activity on February 15, 2021, then recovered to the normal state with a few fluctuations on or about February 22, 2021. According to the experience of a resident living in this census tract during the winter storm, the power outage began on February 15, 2021, which coincided with the greatest dip in activity (Fig. 3). The power recovery occurred intermittently from February 17 to February 19, 2021, which matched fluctuations (Fig. 3). On and after February 20, 2021, as power was fully recovered, activity level returned to normal, evident in the significant jump in activity density between February 19 and 20, 2021.

**Fig. 4:**
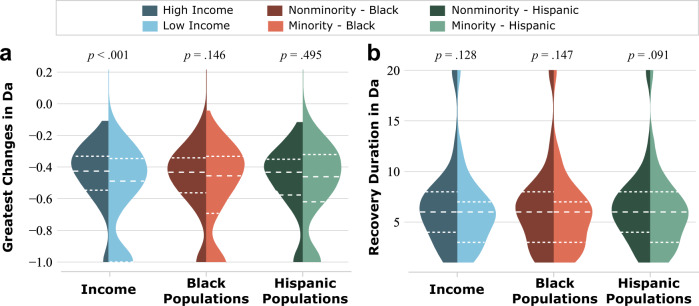
Distribution of the two assessment features of activity density in terms of the income, race, and ethnicity. The two assessment features includes **a** the greatest changes and **b** recovery duration. Results show that census tracts having more low-income or minority groups were experiencing significant impacts in terms of the extent (more negative changes) and duration (longer recovery duration) of the power outages than census tracts having high-income and nonminority groups.

In this study, two features of activity density, the greatest negative changes and recovery duration, were examined to assess the impact of power outages during the storm event. Greatest negative changes indicate the extent of the impact in each census tract. The recovery duration indicates the length of the impact. Figure 4 shows the distribution of both features of activity density in terms of the income, race, and ethnicity. An activity density of −1 for the greatest negative changes feature indicates that those census tracts recorded very little activity, with the implication being that those census tracts suffered significant power outage impacts. On the other hand, some census tracts achieved a recovery duration activity density of 20, an assigned value indicating that those census tracts did not return to the previous normal state in terms of the population activity level before the end of the assessment period.

According to the test results of the six pairs of the minority and nonminority subpopulations (in terms of income, ethnicity, and race) in two different assessment features: greatest activity density change and recovery duration (Fig. 4), the median values of ethnic minority populations in the recovery duration show considerable differences at the significance level of *p* = 0.10. Furthermore, the median values of low-income populations in greatest activity density change are significantly different at the significance level of *p* = 0.01. These results indicate that low-income populations tended to experience greater impacts, and the ethnic minority groups (areas with a high ratio of Hispanic population) had a longer recovery time from power outages. Although there was no statistical difference in the greatest activity density changes between the minority and nonminority groups characterized by race and ethnicity, the median values of the minority groups are both lower than the nonminority groups. Moreover, the number of significantly impacted census tracts in terms of the greatest changes (i.e., the census tracts with 100% decline in population activity levels) for low-income and racial/ethnic minority groups was greater than the number of significantly impacted census tracts for high-income and racial/ethnic nonminority groups. This result implies that more minority census tracts were experiencing more significant outages and subsequent impacts than nonminority census tracts. On the other hand, with respect to recovery duration, the median value for both low-income and racial/ethnic minority categories was six days. In addition, statistical test results indicate no significant difference in terms of the distribution of income levels and racial minorities for recovery duration. Therefore, the next step of the analysis focused on the greatest negative changes in activity density to further reveal the impact of power outages on minority and nonminority groups.

**Fig. 5:**
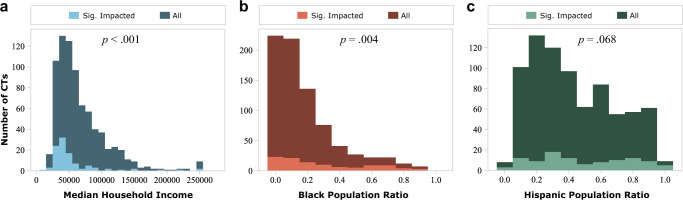
Comparison between all census tracts (CTs) and significantly impacted census tracts due to power outages in Harris County from the aspects of income, race, and ethnicity. The aspects of income, race, and ethnicity are represented by **a** median household income, **b** Black population ratio, and **c** Hispanic population ratio.

Of the 786 census tracts in Harris County, about 13.5% (106) were significantly impacted by power outages, identified by very low population activities. Figure 5 depicts the comparison between all census tracts in Harris County and the significantly impacted census tracts from low-income and racial/ethnic minority groups. The median household income of the significantly impacted census tracts is $40,853 USD, which is lower than the $56,429 USD median income of Harris County. The median ratio of persons identifying as Black in the significantly impacted census tracts is 20.43%, which is higher than the 12.01%, the Harris County median. The median ratio of persons identifying as Hispanic in the significantly impacted census tracts is 43.58%, which is higher than the 38.01% the Harris County median. Statistical tests between all census tracts in Harris County and the significantly impacted census tracts from low-income and racial/ethnic minority groups indicate that the median values between the three pairs are all significant at a significance level of *p* = 0.10. In addition, the *p*-values of the low-income and racial minority groups are both less than 0.05. Thus, based on the results, the significantly impacted census tracts are more likely to include those with a greater proportion of lower income and racial/ethnic minority residents rather than higher-income, nonminority tracts. These results indicate social inequality in the implementation managed power outage during the Texas winter storm.

Despite the results showing that the higher the percentage of minority population, the more likely a census tract is to be more strongly impacted, exceptions always exist. A census tract with a median household income of $32,285 USD, Hispanic population ratio of 94.15%, and Black population ratio of 4.2% with 30.07% of races other than White and Black, was not impacted, according to population activity changes. The activity density of this tract was never lower than that of its pre-disaster normal states. In contrast, a census tract with median household income of $226,602 USD, White population of 94.29%, and non-Hispanic population of 95.08%, had about a 40% decrease in activity density, indicating that the census tract experienced impacts due to power outages. Although the fact that the exceptions exist, census tracts having low-income and racial/ethnic minority groups tend to experience greater impacts due to the greater extent and duration of the power outages.

### Impact of burst pipes

**Fig. 6:**
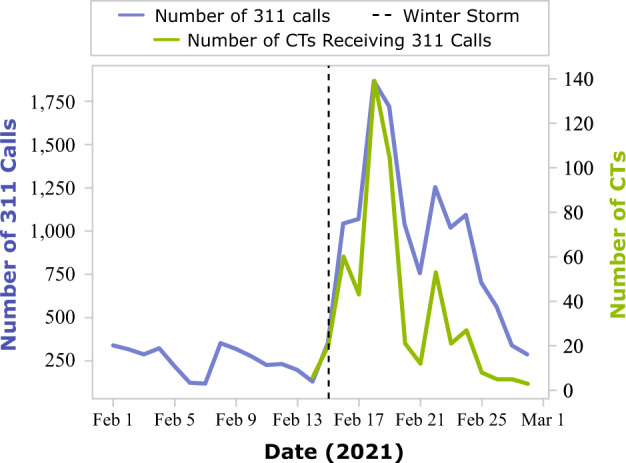
Numbers of the 311 water-related calls (blue) and numbers of census tracts receiving the most water-related calls (orange) during February 2021. Numbers of water-related 311 calls surged after the winter storm reached indicating Harris County underwent burst-pipe issues due to freezing temperatures.

To analyze the condition of water services, this study filtered 311 Houston Service Helpline call data reporting burst pipes based for requested service types inclusive of water leaks, flooding, potable water availability, and poor drainage. Owing to very low 311 call volume in pre-disaster periods, this study conducted analyses with the volume of 311 calls instead of calculating percent changes to baseline periods. Figure 6 indicates a noticeable spike in 311 water-related case numbers during the impact period of the winter storm. High volumes of 311 water-related calls in a census tract may indicate burst-pipe issues due to freezing temperatures. During the impact period of the winter storm, about 67% of the census tracts in Harris County registered water-related 311 cases. Figure 6 also shows the numbers of census tracts receiving the most 311 calls (or case peaks) on each date. The number of water-related cases for most census tracks peaked on February 18 and 19, 2021.

**Fig. 7:**
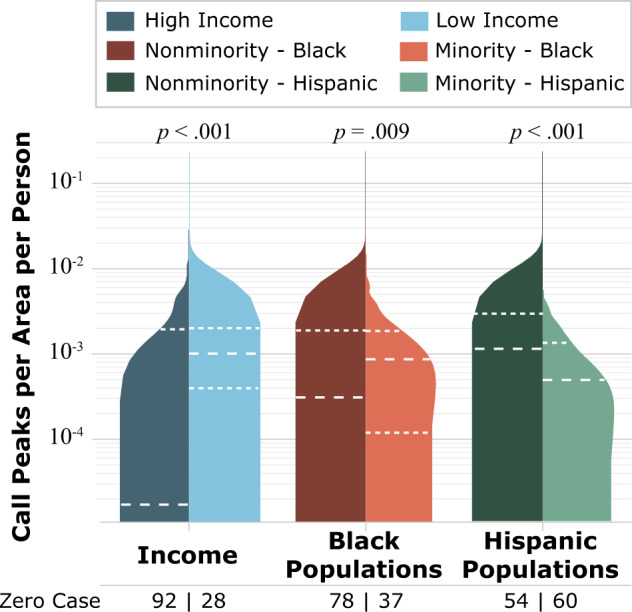
Distribution of the 311 call peaks per area per person for income and race/ethnicity groups and numbers of unimpacted census tracts. Census tracts having more low-income and racial minority groups tend to have higher call peaks per area per person and less unimpacted census tracts.

Multiple analyses were conducted to understand the impact of burst pipes in minority categories; however, differences in population and area may result in a misunderstanding of data. The greater the population in a census tract, statistically, the more 311 calls will originate from that census tract. Furthermore, a larger census tract area would contain more pipes. To account for the variance among census tracts with respect to population and area, the case peaks of each census tract were normalized by dividing by their population and area. Figure 7 shows the distribution of the case peaks per area per person for the low-income and racial/ethnic minority groups. The *p*-values of the statistical test of the differences between the high- and low-income groups and the racial/ethnical minority and nonminority groups are less than 0.05, indicating that the medians of each pair are significantly different. Based on the results, the low-income and racial minority groups had higher normalized case peaks than high-income and racial nonminority groups. Also, given that a census tract receiving zero 311 calls indicates that a census tract is not impacted by burst pipes, twice as many high-income and racial nonminority census tracts were unimpacted compared with low-income and racial minority groups. That low-income and racial minority groups tend to make more 311 calls than the high-income and racial nonminority groups indicates they may suffer more from critical burst pipelines.

**Fig. 8:**
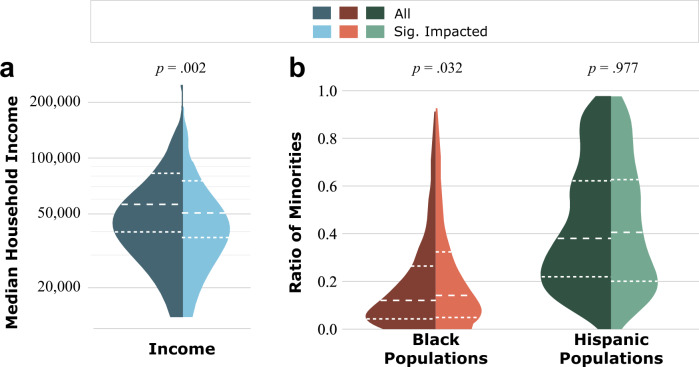
Comparison between all census tracts and the impacted census tracts due to pipe bursts in Harris County from the aspects of income, race, and ethnicity. The aspects of income, race, and ethnicity are represented by **a** median household income, **b** Black and Hispanic population ratio. The impacted census tracts have a lower median income and higher median ratios of Black and Hispanic populations, indicating that the low income and high ratio of Black populations census tracts experienced greater impacts of pipe bursts during the winter storm.

A comparison between all census tracts and the impacted census tracts in Harris County (Fig. 8) revealed that for low-income and racial minority groups, the differences between all census tracts and impacted census tracts are statistically significant at the significance level of *p* = 0.05. The median income of Harris County is $56,429 USD, whereas the median income of the impacted census tracts is $50,589 USD. Also, the median percentage of persons identifying as Black within Harris County is 12.01%; in impacted census tracts is, it is 14.13%. Thus, the census tracts impacted by the burst pipes tend to have lower household income and a higher ratio of Black population.

Overall, the 311 data spike indicated 67% of the census tracts requested maintenance on water-related problems. The comparison between all census tracts and the impacted census tracts shows that the impacted census tracts have lower median household income and a higher ratio of Black population. Thus, low income and high percentage of Black population census tracts experienced more burst pipes during the winter storm. This result indicates the disparities in the experience of extreme weather conditions, such as the winter storm, on vulnerable populations (e.g., low-income and racial minority groups).

### Impact of food inaccessibility

**Fig. 9:**
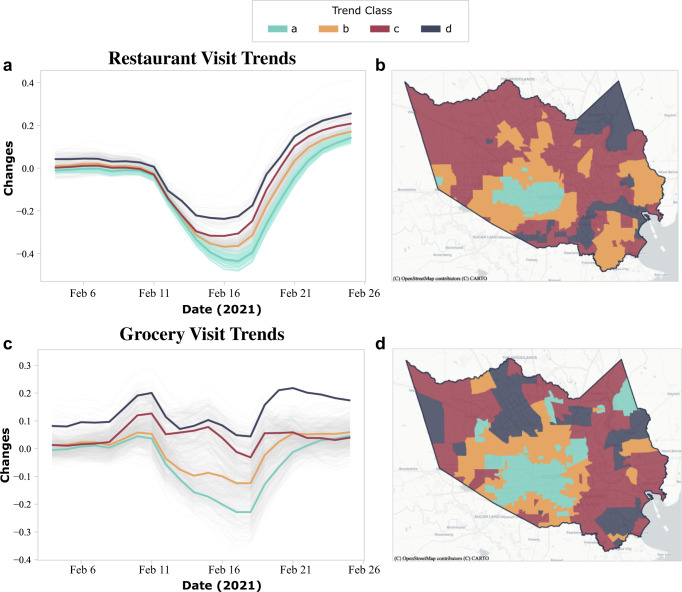
Spatiotemporal distribution of the restaurant and grocery store visits in Harris County. The results of the restaurant visits are shown in **a** and **b**, and the results of grocery store visits are shown in **c** and **d**. Four classes, from Class ***a*** through Class ***d***, represent four patterns of visit trends from the most impacted class to the least impacted class, respectively. Trend line is the average of a group of census tracts having similar patterns and represents the visit fluctuations of these census tracts. The most impacted areas (Class ***a***) of the restaurant and grocery store visits are close to Houston’s downtown areas.

This study obtained data of grocery store and restaurant visits from SafeGraph to reveal fluctuations in food accessibility during the winter storm. Unlike the relationship between activity density and power outages, the food accessibility of a census tract has connections with adjacent census tracts. For example, a resident of one census tract may prefer to buy groceries from a store in a neighboring census tract. Therefore, this study adopted the agglomerative hierarchical clustering algorithm to classify visit trends of census tracts. The advantage of applying this algorithm is that census tracts having similar visit trends can be discovered and grouped to understand the impact of food inaccessibility. Figure 9 shows the classification results of the restaurant and grocery visits in Harris County for every census tract. The census tracts in Harris County are split into four classes. The trend of each class is represented by the mean of all grouped census tracts. The four classes, from ***a*** through ***d***, represent four patterns of visit trends, from the most impacted class to the least impacted class, respectively. An example trend line of class ***a*** (Fig. 9a) is the average of the grouped census tracts (the lighter lines behind trend line of class ***a***) and represents the visit fluctuations of class ***a***.

The study classified different visit trends for the restaurants and grocery stores in Harris County and presented the spatial distribution of each visit trend in Fig. 9. For both types of POIs, class ***a*** represents the most impacted census tracts in terms of visitor numbers; class ***d***, the least impacted census tracts. For restaurant visit trends, the shapes of the four classes are almost identical except for the magnitude of the impact level (Fig. 9a). Visit trends started to decrease on February 11, 2021, and showed the most significant dips February 16 through February 17, 2021. On the other hand, the shapes of the grocery store visit trends (Fig. 9c) are not perfectly identical but demonstrate similar patterns during the winter storm. The grocery store visits had one major and one minor increase before the storm (February 15, 2021), indicative of the preparedness prior to the onset of the winter storm. The largest dips in visits for the grocery stores were on February 18, 2021; they returned to previous normal states afterward.

Spatial autocorrelation analysis was conducted to understand the spatial dependency of the classification results. The Moran’s *I* values for the impact of accessing restaurants and grocery stores were 0.83 and 0.81, and both are significant at the significance level of *p* = 0.01. That is, the spatial dependency exists in the classification results for the impact of accessing both restaurants and grocery stores. Overall, the locations of the most impacted areas of both grocery store and restaurant points of interest are close to Houston’s downtown areas, which is to be expected since the downtown areas are primarily where residents people work rather than their domicile. Owing to the weather and traffic, a population would tend to avoid traveling to work and thus make to fewer visits to business areas compared to the other areas. In addition, most of the secondary impacted areas (class ***b***) are census tracts that surround census tracts identified as class ***a*** (Fig. 9b and 9d). By evaluating the spatial distribution of the visit trends, the most impacted regions in terms of food inaccessibility can be identified in areas surrounding the inner part of Harris County.

**Fig. 10:**
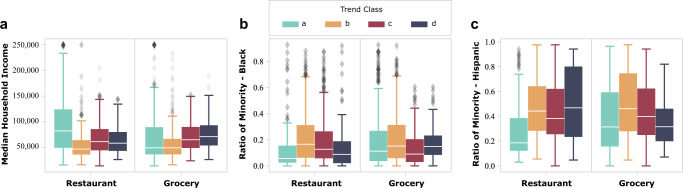
Relationships between food inaccessibility classes and income and racial/ethnic minority groups. The income and racial/ethnic minority groups are based on **a** median household income, **b** Black population ratio, and **c** Hispanic population ratio. Results show that the secondary impacted census tracts generally have lower median income and higher median ratios of Black and Hispanic populations.

In the next step, the relationships between food inaccessibility and the three subpopulation categories were evaluated. Low-income households that rely on public transportation for grocery shopping, for instance, might experience food inaccessibility when public transportation services are suspended. Figure 10 shows the relationship between the four food inaccessibility classes and the three minority categories. The most impacted census tracts (census tracts identified as class ***a***) regarding restaurant and grocery store visits tend to have larger nonminority populations than the other three classes (classes ***b***, ***c***, and ***d***). This result may be due to the fact that the class ***a*** census tracts are primarily located in the downtown area of Houston, which attracts a more affluent population. Owing to a reduced downtown workforce during the storm, visits to restaurants and grocery stores experienced decreased. On the other hand, the median income for the secondary impacted census tracts (census tracts identified as class ***b***) is relatively lower than the other three classes (classes ***a***, ***c***, and ***d***). The median ratio of Black and Hispanic populations for the secondary impacted census tracts (class ***b***) is somewhat higher than the other three classes (classes ***a***, ***c***, and ***d***). This result indicates that those census tracts classified as the secondary impacted classes have a lower income and a higher ratio of Black and Hispanic populations. Therefore, the census tracts with low-income and racial/ethnic minority groups were likely to have food accessibility issues.

## Discussion

This study used the impact of Texas Winter Storm Uri in February 2021, an unusual extreme weather event, in Harris County as a case study. Critical infrastructures, such as power-generating equipment, highways, and the water distribution system, were not well-prepared to withstand a winter storm of Uri’s magnitude. During this critical situation, power-generation plant operators were forced to interrupt services to avoid severely damaging the entire power grid. For example, because power demand increased due to the load imposed by home heating, and power supply decreased due to unwinterized power equipment, ERCOT needed to implement rotating power outages to protect the power grid. It is vital to understand the extent to which these managed power outages were done in an equitable way for different subpopulations. To assess such potential disparate impacts, authors made a concerted effort to obtain granular and high-resolution power outage data by contacting respectable organizations. Yet much data was not publicly available at the time the authors analyzed the issues. In this case, lack of publicly available data provides an opportunity to demonstrate the value of using this community-scale big data as a proxy for actual fine-grained data from the service provider for rapid impact assessments.

In this study, community-scale big data from various sources were used to quantify and assess the spatial patterns of the responses and impacts of the winter storm. This study harnessed population activity data as a proxy to assess the impact of power outages. By leveraging the 311 service helpline records and point-of-interest data, we assessed the impact of burst pipes and food inaccessibility. The quantified impacts of managed power outages, burst pipes, and food inaccessibility were then applied to examine the extent of disparities in impacts experienced by low-income and racial/ethnic minority subpopulations at the census-tract level. Compared with using social media data, which relies on a relatively small portion of geo-coded posts, this study shows digital trace big data that provides geospatial information and reveals population activity serves as a reliable proxy to understand the extent of impacts.

## Closing remarks

Results in all three aspects—impacts of power outages, pipe bursts, and food inaccessibility—indicate the disparities in the experience of extreme weather conditions on vulnerable populations. For power outages and food inaccessibility, results show that census tracts having larger percentages of low-income and racial/ethnic minority groups tend to have been more strongly disrupted by the winter storm. Also, the impact assessment results of burst pipes demonstrate that low-income and racial minority groups are more heavily disrupted than high-income and racial nonminority groups. The results and the statistical comparisons shown in this study reveal only the disparate impacts during the Texas Winter Storm, and we make no implication to any causal inferences. While minority and low-income populations may be particularly susceptible to the impacts of service disruptions, other factors, such as aging infrastructure, may also play a role. Yet, it is still essential to include the consideration of social vulnerability when making decisions for such extreme weather events.

Impacts from the three aspects show different concerns regarding urban disaster resilience in preparedness and response phases. (1) Impacts of power outages in this winter storm indicate how infrastructure operators respond to an unusual event. Although ruggedization of power equipment cannot be accomplished in a short timeframe, the infrastructure operator can make plans for extreme weather scenarios within the considerations of social vulnerability prior to a disaster and inform residents about predetermined hours or days that each area could expect outages when a disaster reaches. In this way, populations can be better prepared. (2) Impacts of burst pipes indicate limited awareness and preparedness of infrastructure owners and residents. Given that temperatures below freezing were predicted, actions could be taken to protect pipes. (3) Impacts of food inaccessibility indicate the influences of service disruptions of critical infrastructures, such as public transportation, road networks, grocery stores, and restaurants.

The disparate impacts revealed in this study can raise awareness in infrastructure managers to take equitable resilience seriously. Service providers should prepare mitigation plans for emergency response and account for these revealed disparities. Rapid assessment results in this study demonstrate that community-scale big data can serve as a tool for local agencies to understand the impact of past severe weather events so that a more equitable allocation of resources can be planned before the arrival of next disaster. To make data comparable with census survey data to understand the existence of disparate impacts, this study aggregated and analyzed data at the census-tract level. The effects of aggregation scale, as known as the Modifiable Areal Unit Problem (MAUP), were not investigated in this study and may require further study to address this concern. Given that the fine-resolution power outage data may become available in the future, results could be further verified and discussed.

## Data Availability

The data that support the findings of this study are available from Mapbox, SafeGraph, and 311, but restrictions apply to the availability of these data, which were used under license for the current study. The data can be accessed upon request submitted on Mapbox and SafeGraph. Other data we use in this study are all publicly available.

## References

[CR3] Almasy S, Silverman H, J Sutton J (2021) More than 150 million Americans under winter weather alerts as record cold temps make life miserable. CNN, Feb 16, 2021

[CR4] Bolin B, Kurtz LC (2018) Race, class, ethnicity, and disaster vulnerability. In: Rodríguez H, Donner W, Trainor JE (eds.) Handbook of Disaster Research Springer International Publishing, Cham, pp. 181–203

[CR5] Cappucci M (2021) 133-Car Pileup on fort worth highway during freezing rain leaves at least 6 dead. Washington Post, Feb 11, 2021

[CR6] Coleman N, Esmalian A, Mostafavi A (2020) Equitable resilience in infrastructure systems: empirical assessment of disparities in hardship experiences of vulnerable populations during service disruptions. Nat Haz Rev 21(No. 4):04020034. 10.1061/(ASCE)NH.1527-6996.0000401

[CR7] Coronese M, Lamperti F, Keller K, Chiaromonte F, Roventini A (2019). Evidence for sharp increase in the economic damages of extreme natural disasters. Proc Natl Acad Sci USA.

[CR8] Day WHE, Edelsbrunner H (1984). Efficient algorithms for agglomerative hierarchical clustering methods. J Classif.

[CR9] Donner W, Rodríguez H (2008). Population composition, migration and inequality: the influence of demographic changes on disaster risk and vulnerability. Soc Forces.

[CR10] Elliott JR, Pais J (2006). Race, class, and hurricane katrina: social differences in human responses to disaster. Soc Sci Res.

[CR11] Fan C, Mostafavi A (2019). A graph-based method for social sensing of infrastructure disruptions in disasters. Computer-Aided Civ Infrastruct Eng.

[CR12] Fan C, Wu F, Mostafavi A (2020). A hybrid machine learning pipeline for automated mapping of events and locations from social media in disasters. IEEE Access.

[CR13] Fan C, Esparza M, Dargin J, Wu F, Oztekin B, Mostafavi A (2020b) Spatial biases in crowdsourced data: social media content attention concentrates on populous areas in disasters. Comput Environ Urban Syst 83:101514. 10.1016/j.compenvurbsys.2020.101514

[CR14] Fan C, Zhang C, Yahja A, Mostafavi A (2021) Disaster city digital twin: a vision for integrating artificial and human intelligence for disaster management. Int J Inform Manag. 56:102049. 10.1016/j.ijinfomgt.2019.102049

[CR15] Farahmand H, Wang W, Mostafavi A, M Maron M (2022) Anomalous human activity fluctuations from digital trace data signal flood inundation status. Environ Plan B: Urban Anal City Sci. 23998083211069990. 10.1177/23998083211069990

[CR51] “Federal Emergency Management Agency.” (2022) Declared disasters. https://www.fema.gov/disaster/declarations. Accessed May 10, 2022

[CR16] Ferman M (2021) Winter storm could cost texas more money than any disaster in state history. The Texas Tribune, Feb 25, 2021

[CR17] Fothergill A, Maestas EGM, Darlington JD (1999). Race, ethnicity and disasters in the United States: a review of the literature. Disasters.

[CR18] Freedman A, Muyskens J, Samenow J (2021) Central states’ Arctic plunge: the historic cold snap and snow by the numbers. Washington Post, Feb 24, 2021

[CR19] Gao X, Fan C, Yang Y, Lee S, Li Q, Maron M, Mostafavi A (2021) Early indicators of human activity during covid-19 period using digital trace data of population activities. Front Built Environ 6. 10.3389/fbuil.2020.607961

[CR20] Hastie T, Tibshirani R, Friedman J (2008) The Elements of Statistical Learning: Data Mining, Inference, and Prediction. Springer

[CR21] Hellerstedt, J (February 2021*)* Winter Storm-Related Deaths—Texas. Texas Department of State Health Services, 2021

[CR22] Hong B, Bonczak BJ, Gupta A, Kontokosta CE (2021). Measuring inequality in community resilience to natural disasters using large-scale mobility data. Nat Commun.

[CR23] Juhasz L, Hochmair H (2020) Studying spatial and temporal visitation patterns of points of interest using safegraph data in Florida. GIS Center. 10.1553/giscience2020_01_s119

[CR24] Kim J, Hastak M (2017) Online human behaviors on social media during disaster responses. J NPS Center Homeland Defense Security. Homeland Security Affairs

[CR25] Kryvasheyeu Y, Chen H, Obradovich N, Moro E, Van Hentenryck P, Fowler J, Cebrian M (2016) Rapid assessment of disaster damage using social media activity. Sci Adv 2(No. 3):e1500779. 10.1126/sciadv.1500779 10.1126/sciadv.1500779 PMC4803483 27034978

[CR26] Li Q, Tang Z, Coleman N, Mostafavi A (2021). Detecting early-warning signals in time series of visits to points of interest to examine population response to COVID-19 pandemic. IEEE Access.

[CR27] Li Q, Bessell L, Xiao X, Fan C, Gao X, Mostafavi A (2021b) Disparate patterns of movements and visits to points of interest located in urban hotspots across US metropolitan cities during COVID-19. Royal Society Open Science 8(No. 1):201209. 10.1098/rsos.201209 10.1098/rsos.201209 PMC7890478 33614069

[CR28] Lu L, Wang X, Ouyang Y, Roningen J, Myers N, Calfas G (2018). Vulnerability of interdependent urban infrastructure networks: equilibrium after failure propagation and cascading impacts: vulnerability of interdependent urban infrastructure networks. Comput-Aided Civ Infrastruct Eng.

[CR29] McCullough ED, McGee K, McCullough J (2021) Texas leaders failed to heed warnings that left the state’s power grid vulnerable to winter extremes, experts say. The Texas Tribune. https://www.texastribune.org/2021/02/17/texas-power-grid-failures/. Accessed Jul 28 2021

[CR30] Mitsova D, Esnard A-M, Sapat A, Lai BS (2018). Socioeconomic vulnerability and electric power restoration timelines in Florida: the case of hurricane Irma. Nat Hazard.

[CR31] Morss RE, Wilhelmi OV, Meehl GA, Dilling L (2011). Improving societal outcomes of extreme weather in a changing climate: an integrated perspective. Ann Rev Environ Resour.

[CR32] Morstatter F, Pfeffer J, Liu H, K Carley K (2013) Is the sample good enough? Comparing data from Twitter’s streaming API with Twitter’s firehose. Proceedings of the International AAAI Conference on Web and Social Media, vol. 7, No. 1

[CR33] Mostafavi A (2018). A system-of-systems framework for exploratory analysis of climate change impacts on civil infrastructure resilience. Sustain Resilient Infrastruct.

[CR34] Mulcahy S (2021) At least 111 people died in Texas during winter storm, most from Hypothermia. The Texas Tribune, Mar 25 2021

[CR52] National Oceanic and Atmospheric Administration” (2022) Winter weather types. NOAA National Severe Storms Laboratory. https://www.nssl.noaa.gov/education/svrwx101/winter/types/. Accessed May 10 2022

[CR35] Panteli M, Mancarella P (2015). Influence of extreme weather and climate change on the resilience of power systems: impacts and possible mitigation strategies. Electr Power Syst Res.

[CR36] Pasupathi S, Shanmuganathan V, Madasamy K, Yesudhas HR, Kim M (2021). Trend analysis using agglomerative hierarchical clustering approach for time series big data. J Supercomput.

[CR37] Peacock WG, Zandt SV, Zhang Y, Highfield WE (2014). Inequities in long-term housing recovery after disasters. J Am Plan Assoc.

[CR38] Podesta C, Coleman N, Esmalian A, Yuan F, Mostafavi A (2021) Quantifying community resilience based on fluctuations in visits to points-of-interest derived from digital trace data. J R Society Interf 18:177. 10.1098/rsif.2021.0158 10.1098/rsif.2021.0158 PMC8086905 33906388

[CR39] Podlaha A, Bowen S (2014). February 2014 Global Catastrophe Recap. Aon Benfield

[CR40] Ragini JR, Anand PMR, Bhaskar V (2018). Big data analytics for disaster response and recovery through sentiment analysis. Int J Inform Manag.

[CR41] Ray A, Camiolo M, Fitzpatrick A, Gauthier M, Wenzel SE (2020). Are we meeting the promise of endotypes and precision medicine in asthma. Physiol Rev.

[CR42] Reid M (2013). Disasters and social inequalities. Sociol Compass.

[CR43] Resch B, Usländer F, Havas C (2018). Combining machine-learning topic models and spatiotemporal analysis of social media data for disaster footprint and damage assessment. Cartogr Geogr Inform Sci.

[CR44] Rubin GJ, Rogers MB (2019) Behavioural and psychological responses of the public during a major power outage: a literature review. Int J Disaster Risk Reduc 38:101226. 10.1016/j.ijdrr.2019.101226

[CR45] Steiger BK, Kegel LC, Spirig E, Jokeit H (2019). Dynamics and diversity of heart rate responses to a disaster motion picture. Int J Psychophysiol.

[CR46] Stocker T (ed.) (2014) Climate Change 2013: The Physical Science Basis: Working Group I Contribution to the Fifth Assessment Report of the Intergovernmental Panel on Climate Change. Cambridge University Press, New York

[CR47] Watson KP, Cross R, Jones MP, Buttorff G, Pinto P, Sipole SL, Vallejo A (2021) The effects of the winter storm of 2021 in Harris County. Hobby School of Public Affairs, University of Houston

[CR48] Xiao Y, Huang Q, Wu K (2015). Understanding social media data for disaster management. Nat Hazards.

[CR49] Yuan F, Yang Y, Li Q, Mostafavi A (2022). Unraveling the temporal importance of community-scale human activity features for rapid assessment of flood impacts. IEEE Access.

[CR50] Zhang C, Yao W, Yang Y, Huang R, Mostafavi A (2020). Semiautomated social media analytics for sensing societal impacts due to community disruptions during disasters. Comput-Aided Civ Infrastruct Eng.

